# Recognizing Signs of Abusive Head Trauma

**DOI:** 10.1111/jpc.70449

**Published:** 2026-05-25

**Authors:** Maísa Naomi Irie, Vinicius de Menezes Jarry, Matheus de Lima Ruffini, Adolfo Moraes de Souza, Mariana Dalaqua, Juliana Ávila Duarte, Andrea Fraga, Fabiano Reis

**Affiliations:** ^1^ Department of Radiology Universidade Estadual de Campinas (Unicamp) Campinas SP Brazil; ^2^ Department of Radiology and Diagnostic Imaging Hospital de Clínicas de Porto Alegre (HCPA) Porto Alegre RS Brazil; ^3^ Neuchâtel Hospital Network (RHNe) Neuchâtel Switzerland; ^4^ Department of Pediatrics Universidade Estadual de Campinas (Unicamp) Campinas SP Brazil

**Keywords:** abusive head trauma, battered child syndrome, child abuse, craniocerebral trauma, neuroimaging, paediatric radiology, shaken baby syndrome

## Abstract

**Background:**

Abusive head trauma (AHT) remains the leading cause of fatal traumatic brain injury in infants and young children. Diagnosis is frequently delayed or missed due to nonspecific clinical presentation and unreliable histories, making neuroimaging a critical component of the diagnostic process.

**Objective:**

This review synthesises computed tomography (CT) and magnetic resonance imaging findings (MRI) associated with abusive mechanisms and highlights patterns that may support clinical suspicion, multidisciplinary decision making and child protection interventions.

**Methods:**

A narrative review of characteristic imaging features in AHT was conducted, focusing on the radiologic appearance of retinal haemorrhages, subdural hematomas, subarachnoid and epidural haemorrhages, bridging vein thrombosis, spinal subdural haemorrhages, hypoxic–ischemic damage, parenchymal lesions and skull injuries.

**Results:**

Subdural hematoma was the most frequently reported imaging finding, often bilateral or asymmetric, typically involving the high convexities, the interhemispheric fissure and the posterior fossa. Additional findings commonly associated with AHT included retinal haemorrhages, bridging vein thrombosis, spinal subdural haemorrhage, HII injury, hemispheric hypodensity, traumatic axonal injury and complex skull fractures. MRI demonstrated superior sensitivity compared with CT for detecting small haemorrhages, early parenchymal injury and axonal damage.

**Conclusion:**

Recognition of characteristic neuroimaging patterns plays a central role in the evaluation of suspected AHT. Early identification by radiologists and paediatric clinicians is critical not only for guiding acute management but also for activating multidisciplinary child protection responses and preventing recurrent injury, long‐term neurological impairment and fatal outcomes.

## 1 Introduction

Child abuse is a major global health issue. According to the World Health Organization (WHO), approximately three out of four children aged 2–4 years are victims of physical punishment and/or psychological violence. It is estimated that 40 150 intentional violent deaths of children occur every year [1].

Brain injuries are the leading cause of traumatic death in infants younger than 2 years old. The combination of findings in non‐accidental head trauma was first described as ‘shaken baby syndrome’. Currently recommended nomenclature favours the theme ‘abusive head trauma’ (AHT), which encompasses a broader spectrum of mechanisms, including shaking, blunt impact and combined injury patterns [2].

Traumatic head injuries are not uncommon in the paediatric population. Clinical presentation varies according to the mechanism of trauma, and neurological manifestations are often nonspecific. Common symptoms include altered level of consciousness, seizures, vomiting, irritability and neuropsychomotor developmental delay [3].

Accurate diagnosis of AHT remains challenging and is frequently underestimated. Previous studies have demonstrated that approximately 20% of children who ultimately died as a result of abuse had been evaluated by healthcare professionals prior to death. A thorough review of the reported mechanism of injury and past medical history is essential, as caregiver accounts may be incomplete, inconsistent or misleading. In most cases, abuse is inflicted by parents or primary caregivers [3].

Within this context, neuroimaging plays a pivotal role in the diagnostic evaluation. Although no clinical or radiological finding is pathognomonic for AHT, specific imaging patterns may raise suspicion and support the diagnosis when integrated with clinical and social information [3].

### 1.1 Objective

This study aims to illustrate the principal neuroimaging findings on computed tomography (CT) and magnetic resonance imaging (MRI) that should alert attending physicians, particularly radiologists and paediatricians, to the possibility of AHT.

## 2 Methods

A narrative review was conducted to describe and critically analyse characteristic neuroimaging features associated with AHT. A targeted literature search was performed using combinations of the terms AHT, shaken baby syndrome, non‐accidental injury, CT, MRI, susceptibility‐weighted imaging (SWI), diffusion‐weighted imaging (DWI), subdural haemorrhage, bridging veins, retinal haemorrhage and hypoxic–ischemic injury (HII). Priority was given to higher‐level evidence, including systematic reviews and comparative studies, as well as publications addressing mechanism‐based imaging patterns and radiologic differentiation between abusive and accidental head trauma. Particular emphasis was placed on the diagnostic contributions of CT and MRI, including advanced sequences such as SWI and DWI.

Eligible studies were required to (i) describe CT and/or MRI findings in infants or young children with suspected AHT; (ii) address diagnostic frameworks and differential diagnostic considerations and (iii) include explicit criteria or comparative analyses supporting the distinction between abusive and accidental mechanisms.

Literature was evaluated qualitatively, focusing on consistency of reported imaging patterns, pathophysiological plausibility and integration of radiologic findings with multidisciplinary diagnostic confirmation. Illustrative imaging cases from Brazilian hospital settings were included, all anonymised and used exclusively for educational purposes in accordance with institutional ethical standards.

## 3 Results

### 3.1 Subdural Hematoma

Subdural hematoma (SDH) represents the most frequent intracranial imaging finding in AHT [4]. It commonly involves the high convexities, interhemispheric fissure and posterior fossa (Figures 2c and 3b), and is frequently bilateral and asymmetric (Figure 3a). Determination of the chronological stage of SDHs based on imaging findings can be challenging. MRI is more accurate than CT for staging, particularly for small or chronic collections (Figures 1b,c and 4d) [5].

**FIGURE 1:**
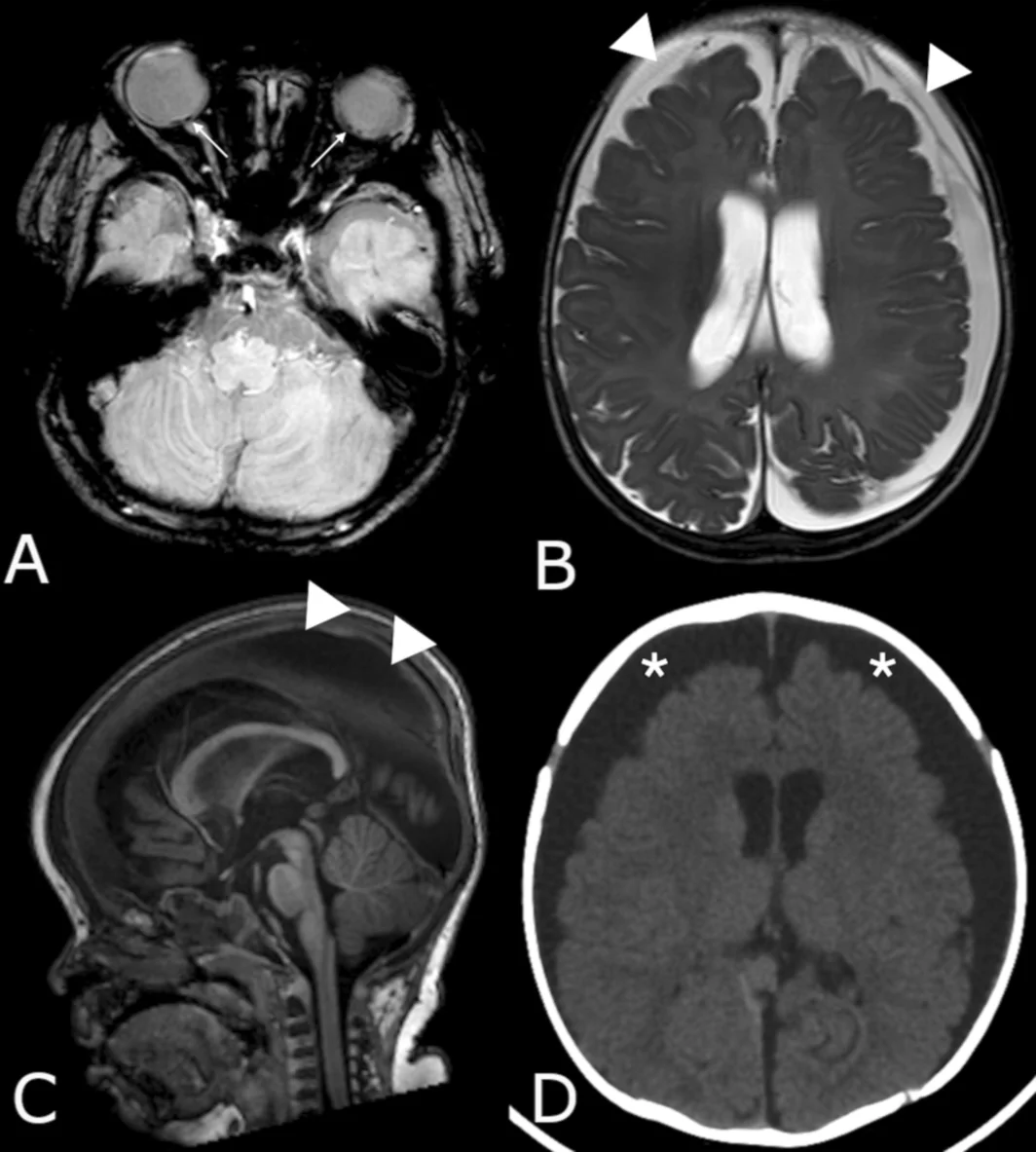
A 5‐month‐old boy was taken to the emergency department due to vomiting and irritability. (a) Axial susceptibility‐weighted MR image (SWI) depicts retinal haemorrhages as hypointense foci along posterior globes (arrows). (b) Bilateral subdural collections, heterogeneous, with high signal on T2‐WI (arrowheads). (c) Sagittal T1‐WI demonstrates a low signal intensity collection in the subdural space along the high convexity (arrowheads). (d) Axial CT image performed after 3 months shows frontotemporal subdural collections with similar attenuation to cerebrospinal fluid (asterisks).

**FIGURE 2:**
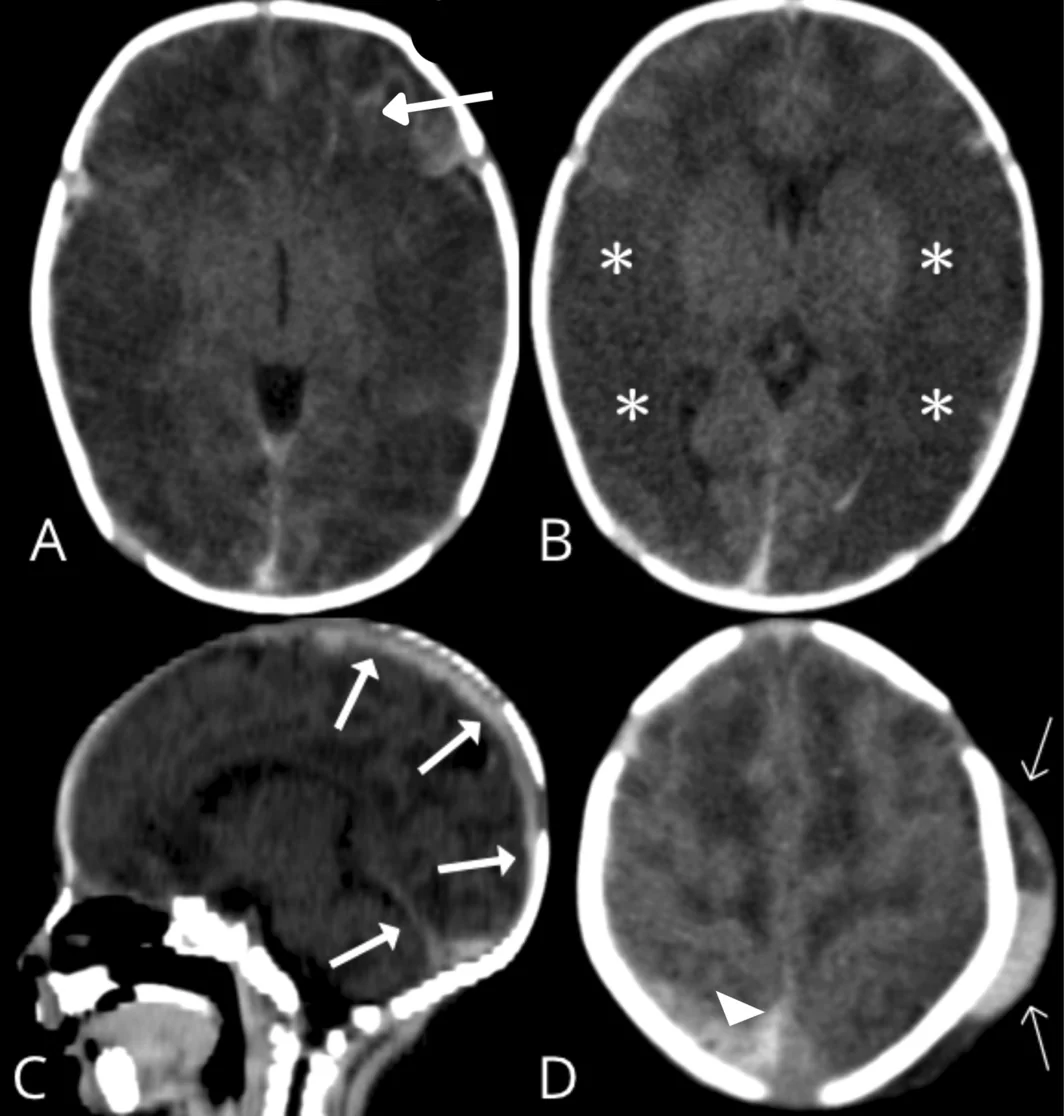
Male newborn presented with bruises, decreased feeding and lethargy and unenhanced head CT images were performed on the day he was taken to the emergency department. (a) Axial image depicts acute subarachnoid haemorrhage filling frontal sulci (arrow). (b) Axial image demonstrates diffuse cortico‐subcortical hypodensities in frontal and temporo‐occipital lobes (asterisks). (c) Sagittal image shows a right acute fronto‐parieto‐temporal subdural hematoma extending to the interhemispheric fissure (three superior arrows) and along the tentorium (inferior arrow). (d) Axial image demonstrates a right parietal acute subdural hematoma (arrowhead) and an acute subgaleal hematoma along the left frontoparietal outer table of the calvaria (arrows).

**FIGURE 3:**
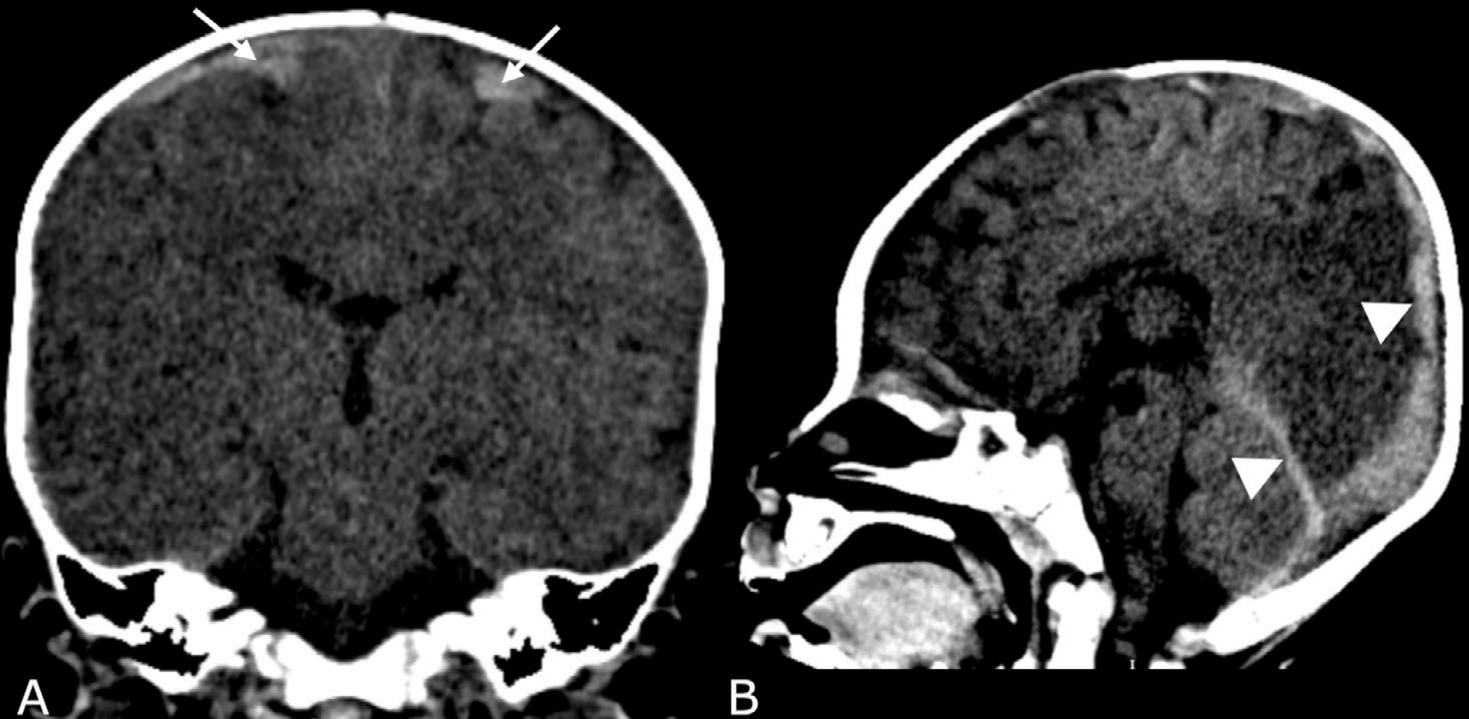
Non‐contrast head CT images of a 5‐month‐old boy with head trauma due to a reported fall from the bed. (a) Coronal plane depicts bilateral hyperdense extra‐axial collections along the high convexity, in keeping with subdural haemorrhage. (b) Sagittal image evidences acute subdural haemorrhages in the interhemispheric fissure (superior arrowhead) and along the tentorium (inferior arrowhead).

**FIGURE 4:**
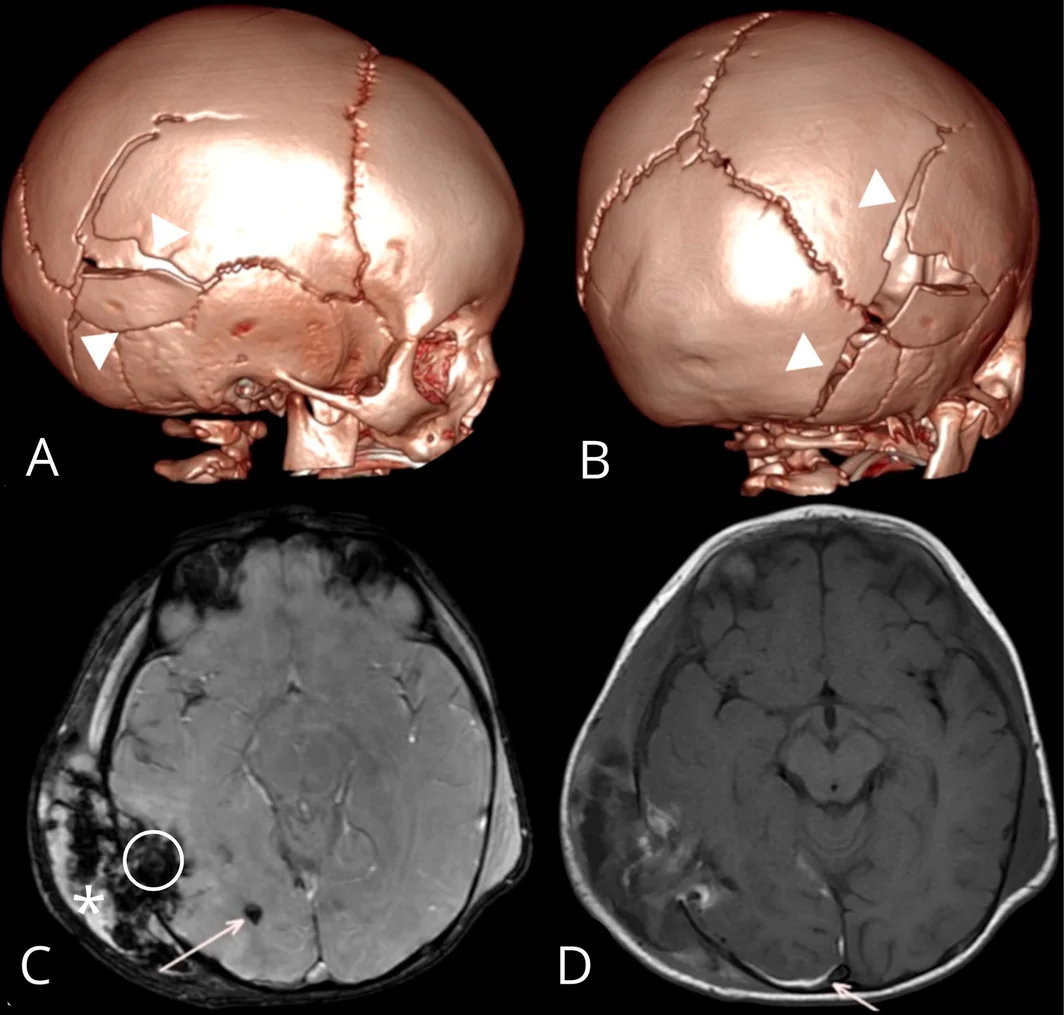
A 10‐month‐old boy who allegedly fell off the couch. Images (a) and (b) are three‐dimensional computed tomography reconstructions showing multiple parietal and occipital bone fractures (arrowheads). (c) Axial SWI demonstrates a hypointense focus in the occipital horn of the lateral ventricle (arrow), as well as a hypointense soft tissue collection along the right temporo‐occipital region of the calvaria, characterising a subgaleal hematoma (asterisk). There is also hypointensity in the right temporo‐occipital parenchyma, consistent with a hemorrhagic contusion (circle). (d) Axial T1‐WI depicts a thin hyperintense right occipital subdural hematoma (arrow).

In the acute phase, SDH typically appear as hyperdense crescent‐shaped collections on non‐contrast CT (Figure 2d). Mixed density collections may be observed in cases of acute on chronic bleeding, sedimentation levels or hemato‐hygromas (when bleeding occurs inside subdural hygromas) [6]. During the late subacute phase, SDH may become isodense relative to the cerebral cortex [7]. Chronic collections usually appear hypoattenuating and may resemble subdural hygromas (Figure 1d). The presence of enhancing inner membranes and dural thickening favours chronic hematoma over hygroma and typically becomes evident 2–3 weeks after injury [6].

On MRI, signal characteristics of subdural haemorrhage vary according to haemoglobin degradation products [8]. In the acute phase, collections are iso‐ to hypointense on T1‐WI, and hypointense on T2‐WI. Subacute hematomas demonstrate hyperintensity on both T1 and T2 weighted images. On SWI, signal appearance varies by phase and depends both on T2* effects and T1 signal intensity of blood products: iso or hyperintensity in the hyperacute phase (first 12–24 h); hypointensity in acute and early subacute phases; central hyperintensity in late subacute hematomas, and a peripheral low signal rim in chronic collections [8].

Benign enlargement of subarachnoid spaces (subdural hygromas) should be considered in the differential diagnosis of chronic SDHs, particularly on CT [4]. Subdural hygroma is an accumulation of cerebrospinal fluid in the subdural space and is a common complication of traumatic brain injury, as indicated by an incidence of 6%–21.6% [9]. In subdural hygromas, cortical vessels traverse the widened subarachnoid space; collections are symmetric, isodense or isointense to cerebrospinal fluid, and there is no associated mass effect. Macrocephaly may be present [6]. However, it should be noted that distinguishing chronic SDH and subdural hygromas, especially on imaging, may be difficult and the terms are often used interchangeably.

### 3.2 Bridging Vein Thrombosis

Bridging veins connect superficial cortical veins to the superior sagittal sinus and traverse both the subarachnoid and subdural spaces. Acceleration, deceleration and rotational forces may result in tearing of these veins, leading to subdural haemorrhage. Thrombosis may subsequently develop within or adjacent to the injured vein [6].

The so‐called ‘tadpole’ or ‘lollipop’ sign consists of an oval or round thrombus representing the body of the tadpole, with an elongated tail corresponding to the torn bridging vein distended by clot. This sign may be identified on CT but is best visualised on MRI, particularly on gradient‐echo (GRE) or SWI sequences. Time‐of‐flight (TOF) MR venography may demonstrate loss of signal or discontinuity along the affected vein [10].

Although not pathognomonic, bridging vein thrombosis supports a traumatic aetiology of SDH [6] and, when associated with additional findings such as retinal haemorrhage, strengthens suspicion for AHT [10].

### 3.3 Retinal Haemorrhage

Retinal haemorrhages are identified in up to 85% of AHT cases [11]. In abusive mechanisms, haemorrhages are typically bilateral, involve multiple retinal layers and extend peripherally towards the *ora serrata*. Severe cases may extend into the vitreous and result in visual impairment.

Although diagnosis is primarily clinical through ophthalmoscopic examination, retinal haemorrhages may occasionally be detected on imaging as hyperattenuating foci along the posterior globes on CT and as low‐signal foci on MRI (Figure 1a). SWI significantly increases sensitivity for detection [7].

### 3.4 Subarachnoid Haemorrhage

Subarachnoid haemorrhage (SAH) is more commonly associated with accidental trauma but has been reported in autopsy studies of fatal AHT [12]. While nonspecific, its presence in association with retinal haemorrhage, SDH and parenchymal injury should raise concern for abuse. On CT, SAH appears as hyperdense material filling sulci, fissures and cisterns. In AHT, it more frequently involves high convexities (Figure 2a). MRI, particularly FLAIR and SWI sequences, demonstrates higher sensitivity than CT [5].

### 3.5 Epidural Hematoma

Epidural hematoma represents blood accumulation between the inner table of the skull and the dura mater, usually resulting from arterial (particularly of the middle meningeal artery) or venous injury [5]. It is less commonly observed in AHT and more frequently associated with accidental mechanisms, such as falls [12]. When present in conjunction with skull fractures, careful correlation with the reported mechanism of injury is warranted [5].

### 3.6 Spinal Subdural Haemorrhage

Spinal subdural haemorrhage has been reported in over 60% of abused children who underwent thoracolumbar imaging, while being uncommon in accidental trauma [13]. These haemorrhages most frequently involve the thoracolumbar region, reflecting both gravitational migration of intracranial blood from the posterior fossa and/or direct injury to vessels in this compartment [14]. Data suggest underutilization of complete spinal imaging, which should be offered for all children with moderate or severe traumatic brain injury [13].

On CT, spinal subdural haemorrhage appears hyperdense relative to surrounding structures. MRI signal characteristics vary with the stage of haemorrhage, with early lesions demonstrating high signal intensity on T1‐WI, remaining, however, less intense than fat. Mass effect on the spinal cord may be present [13].

### 3.7 Hemispheric Hypodensity Associated With SDH

Hemispheric hypodensity describes diffuse cortical and subcortical hypodensity with loss of grey‐white matter differentiation on CT. This pattern may involve one or both hemispheres (Figure 2b) and is commonly associated with SDH (Figure 2c) [15].

Mismatch between oxygen supply and demand, as well as systemic insults impairing oxygen delivery to the brain, may explain the pathogenesis. Affected parenchyma often demonstrates rapid progression to encephalomalacia and atrophy [15]. Mortality approaches 70%, and survivors typically experience severe neurological impairment.

Although hemispheric hypodensity may occur in accidental trauma, it has been more frequently reported in AHT [15].

### 3.8 Hypoxic–Ischemic Injury (HII)

HII represents a major contributor to morbidity and mortality in AHT, but remains under‐recognised even though it may explain various degrees of developmental delay and seizures.

Two predominant patterns have been described: asymmetric cortico‐subcortical injury and diffuse involvement of both superficial and deep grey‐white matter. These patterns suggest mechanisms including central apnea, vascular compromise or strangulation [16].

DWI is essential for early detection of cytotoxic edema and should be included in MRI protocols.

Zimmerman, Bilaniuk and Farina [17] have described two different DWI patterns of injury in children with AHT: diffuse, bilateral supratentorial cortico‐subcortical parenchymal infarction (the most severe form) or watershed infarctions, mostly between middle and anterior cerebral arteries.

Orru et al. [16] have observed a predominance of asymmetric distribution of the lesions, with cortico‐subcortical cytotoxic edema. The mechanism explaining asymmetric HII might be different from that of global cytotoxic edema, with or without involvement of deep grey nuclei. A possible explanation could be that these patients had temporary compromise or occlusion of neck vessels more severe on one side, resulting in a side‐specific occurrence of cytotoxic edema [16].

### 3.9 Other Parenchymal Injuries

Children are particularly susceptible to parenchymal injury due to a larger head‐to‐body ratio, weaker cervical musculature and thinner skull [18]. Parenchymal injuries vary according to the mechanism and intensity of trauma, including contusions, lacerations, traumatic axonal injury (TAI) and intraparenchymal haemorrhage. While nonspecific, these findings are critical for diagnosis and prognosis [19].

Cerebral contusions result from the collision of parenchyma against the inner table of the skull. Most affected regions are inferior frontal and temporal lobes, due to the impact against anterior and middle cranial fossa [6]. They can occur at the site or on the opposite site of the impact (*coup*‐*contrecoup* contusion), or adjacent to a skull fracture. These injuries are rarely observed in infants (less than 5 years old), which is attributable to the compliance and smoothness of their skull. Large contusions can be detected on CT as hyperdense foci, and may be surrounded by low density edema [20]. Small cortical contusions are difficult to depict on CT and are easily detected on MRI with SWI and GRE sequences (particularly hemorrhagic contusions) (Figure 4c) and DWI (in non‐hemorrhagic contusions).

TAI results from rotational and acceleration‐deceleration forces, stretching axons. Although the presence of TAI does not help to distinguish accidental from non‐accidental head trauma, it is very relevant to prognosis [6]. Despite the limited sensitivity of CT, these injuries can appear as petechial hyperdense foci or multiple hypodense foci with diffuse edema in the deep white matter. TAI is best depicted on MRI as multiple hypointense foci on T2*‐GE and SWI sequences (hemorrhagic TAI), variable signal intensity on T1 and/or hyperintense foci on DWI and T2‐WI (non‐hemorrhagic TAI) [20].

Parenchymal lacerations may be observed in AHT. The MRI criteria for diagnosing a laceration include: (1) a parenchymal cleft containing CSF, haemorrhage or a CSF‐haemorrhage fluid level; (2) a linear or oval cleft with a paramagnetic substance demonstrating GRE imaging or SWI hypointensity or a perimeter of diffusion restriction; and (3) a linear signal alteration of > 5 mm (including T2/FLAIR hyperintense signal and/or T1 shortening) that did not correspond to a sulcus or perivascular space.

Lacerations may be a distinct pattern of injury related to AHT and are likely the result of angular acceleration, deceleration and rotational forces that occur when infants have AHT whether through shaking and/or impact or even crushing injuries. So, the radiologist should appreciate the finding of a parenchymal brain laceration as a finding for AHT [21].

Parenchymal injuries, depending on the mechanism and extension of trauma, may progress to encephalomalacia and gliosis, leading to sequellar cavities or cerebral atrophy [20].

### 3.10 Skull Fractures

Skull fractures are not specific for AHT; however, certain patterns may raise concern for high‐energy mechanisms. These include multiple, complex, diastatic (with displacement greater than 3 mm from suture lines), depressed or ‘growing fractures’, and the occipital bone is the most involved in this context [22]. Three‐dimensional CT reconstructions are valuable for characterisation (Figure 4a,b) [23].

Determination of fracture age remains challenging, though associated findings such as subgaleal hematoma may suggest recent injury [19].

## 4 Conclusion

This review highlights the neuroimaging findings most frequently observed in AHT and emphasises their diagnostic importance. Although no single imaging feature is pathognomonic, recognition of characteristic patterns, particularly in the presence of clinical inconsistencies, is essential for early diagnosis. Prompt identification by radiologists and paediatric clinicians plays a critical role in guiding medical management, informing medico‐legal evaluation and activating child protection responses to prevent recurrent injury, long‐term harm and fatal outcomes.

## Funding

The authors have nothing to report.

## Conflicts of Interest

The authors declare no conflicts of interest.

## Data Availability

Data sharing not applicable to this article as no datasets were generated or analysed during the current study.

## Appendix A

### Questions

1. **For a 4‐month‐old boy who presented seizures after falling from the bassinet, what imaging study of the head is required first?**
  - X‐ray
  - **Computed tomography (CT)**
  - Ultrasound
  - Magnetic resonance imaging (MRI)
  - None
2. **Retinal haemorrhages are found in up to 85% of abusive head traumas (AHT). Which of the following statements is *TRUE* about retinal haemorrhages?**
  - In AHT, it is often unilateral and involves one or two retinal layers.
  - The diagnosis is only clinical, with ophthalmoscopic examination.
  - They can be identified as hypoattenuating foci in the posterior portion of the globe at CT.
  - MRI has low sensitivity for these haemorrhages.
  - **SWI sequence shows multiple foci of susceptibility along the posterior surface of the globes**.
3. **Which of the following imaging findings is *LESS* frequent in a non‐accidental trauma:**
  - **Epidural hematoma**.
  - Subdural hematoma.
  - Subarachnoid haemorrhage.
  - Retinal haemorrhages.
  - Traumatic axonal injury.
4. **A non‐contrast head CT shows hyperdensity filling some right frontal sulci. What is the most likely diagnosis?**
  - Subdural hematoma.
  - Epidural hematoma.
  - **Subarachnoid haemorrhage**.
  - Cerebral contusion.
  - Traumatic axonal injury.
5. **All the following statements are true, *EXCEPT*:**
  - **Subdural hematoma represents a blood collection between the inner table of the skull and the dura mater**.
  - The typical localizations of subdural hematoma in abusive head trauma are along high convexities, interhemispheric space, and the posterior fossa.
  - An acute subdural hematoma is classically characterised as a hyperdense collection with a crescent‐shaped morphology on non‐enhanced CT.
  - In the late subacute phase, subdural hematoma appears isodense to the cerebral cortex on CT.
  - Chronic subdural hematomas are usually hypoattenuating and resemble subdural hygromas.
6. **Which CT imaging characteristic helps distinguish chronic subdural hematoma from hygroma?**
  - Localisation.
  - Attenuation like cerebrospinal fluid.
  - **Presence of contrast‐enhancing inner membranes**.
  - Morphology of the collection.
  - Bilateral collections.
7. **Studies demonstrated that spinal subdural haemorrhages were present in more than 60% of abused children who underwent thoracolumbar imaging. Which sentence below is *INCORRECT*?**
  - Spinal subdural haemorrhages are more common in inflicted traumas.
  - **The most affected segment is the cervical spine**.
  - In early stages, it shows high signal on T1‐WI.
  - It is frequently associated with subdural hematoma in the posterior fossa.
  - A complete spinal study should be considered for all children with moderate or severe traumatic brain injury.
8. **About the ‘tadpole’ sign, which affirmation is *FALSE*?**
  - This sign refers to a characteristic morphology of post‐traumatic bridging vein thrombosis.
  - The round shape of the tadpole represents the venous thrombus.
  - The tail of the tadpole reflects the torn bridging vein expanded by thrombosis.
  - **This sign can be identified only by MRI**.
  - The ‘tadpole’ sign is better demonstrated with gradient‐echo (GRE) or SWI sequences.
9. **Which statement below is *TRUE* about traumatic axonal injury (TAI)?**
  - TAI is a neuroimaging finding with high specificity for abusive head trauma.
  - The presence of TAI does not affect prognosis.
  - CT reliably shows multiple hyperdense foci in deep white matter.
  - TAI is best characterised on MRI as multiple hyperintense foci on T2* and SWI sequences.
  - **It is caused by sudden rotational or acceleration and deceleration head movements**.
10. **The most frequently affected bone in non‐accidental head trauma is:**
  - Parietal.
  - **Occipital**.
  - Temporal.
  - Frontal.
  - Sphenoid.
